# Integrated Metabolomics and Proteomics Analyses of the Grain-Filling Process and Differences in the Quality of Tibetan Hulless Barleys

**DOI:** 10.3390/plants14131946

**Published:** 2025-06-25

**Authors:** Yanrong Pang, Yuping Yang, Kaifeng Zheng, Xiaozhuo Wu, Yanfen Zhang, Jinyuan Chen, Guigong Geng, Feng Qiao, Shengcheng Han

**Affiliations:** 1 College of Life Sciences, Beijing Normal University, Beijing 100875, China; 202321200016@mail.bnu.edu.cn (Y.P.); yangyuping19860522@126.com (Y.Y.); kaifeng_zheng@mail.bnu.edu.cn (K.Z.); 2 Key Laboratory of Biodiversity Formation Mechanism and Comprehensive Utilization of the Qinghai-Tibet Plateau in Qinghai Province, School of Life Sciences, Qinghai Normal University, Xining 810008, China; xiaozhuo0623@163.com (X.W.); 13897450796@163.com (Y.Z.); 20211027@qhnu.edu.cn (J.C.); 3 Academy of Agricultural and Forestry Sciences, Qinghai University, Xining 810016, China; genggg-298@163.com; 4 Academy of Plateau Science and Sustainability of the People’s Government of Qinghai Province & Beijing Normal University, Beijing 100875, China, Qinghai Normal University, Xining 810008, China

**Keywords:** metabolomics, proteomics, difference in quality, Tibetan hulless barley

## Abstract

Tibetan hulless barley (qingke) grains are becoming more popular because of their high nutritional benefits. Comparative metabolomics and proteomics analyses of qingke grains (at 16, 20, 36, and 42 days after flowering) were conducted to explore the metabolic dynamics during grain filling and compare the differences in quality among three different varieties, Dulihuang, Kunlun 14, and Heilaoya. A total of 728 metabolites and 4864 proteins were identified. We first found that both the metabolite and protein profiles were more closely associated with the grain developmental stage in each cultivar than across different stages in a single cultivar. Next, we focused on the energy metabolism and biosynthesis pathways of key quality components, such as flavonoids, starch, and β-glucans in qingke grains. Quantitative analysis revealed significant variation in the abundance of cellulose synthase-like enzyme (CslF) among the three cultivars. Notably, Heilaoya displayed substantially lower CslF6 levels at 36 and 42 DAF than Kunlun 14 and Dulihuang did. These observed differences in CslF6 abundance may represent a key regulatory mechanism underlying the distinct β-glucan biosynthesis patterns among the three cultivars. Collectively, our results enhance the understanding of metabolic networks involved in qingke grain development and serve as a foundation for advancing breeding studies.

## 1. Introduction

Barley (*Hordeum vulgare* L.) is one of the earliest cultivated cereals in the Poaceae family and ranks as the fourth most produced cereal globally, following maize (*Zea mays*), wheat, and rice [1]. Hulless barley (*Hordeum vulgare* L. var. *nudum*), known as “Qingke” in Chinese, is the staple grain for Tibetans and a critical feed source for livestock on the Tibetan Plateau [2]. Hulless barley (HB), a member of the grass family, is an annual barley cultivar [3]. The development of barley grains involves a complex biological process that begins with fertilization and continues until physiological maturity. This process typically spans a period of 14 to 40 days, with the duration varying on the basis of factors such as the environmental conditions, genotype, and spatial location of the grain in the head [4,5]. Generally, the process can be categorized into three stages characterized by continuous cell division and morphogenesis (cell division or pre-storage phase), accumulation of storage products (storage or maturation phase), and dehydration (desiccation phase). Additionally, there is an intermediate or transition phase between the pre-storage and storage phases, characterized by significant alterations at the transcriptional level and various physiological parameters [6,7].

Qingke is rich in bioactive compounds, including high concentrations of dietary fiber, vitamins, and soluble β-glucan and low lipid and carbohydrate levels [8,9]. As a non-starch polysaccharide in qingke, β-glucan has shown remarkable physiological benefits, i.e., anti-immunomodulatory and anticancer properties, the inhibition of cholesterol synthesis, the regulation of the glycemic response, and support for weight management, as evidenced by prior studies [10,11]. It is also widely recognized as a rich source of phytochemical derivatives, namely flavonols, phenolic acids, flavones, chalcones, proanthocyanidins, and flavanones. Phenolic compounds are valuable dietary materials offering antioxidant and anti-inflammatory benefits [12,13]. Owing to its abundance of physiologically active compounds, qingke has become increasingly popular as a raw material for functional food production. Starch is the primary storage compound in the endosperm of qingke cereal grains, and its quality characteristics significantly impact the appearance and edible quality of flour-based products [14]. Moreover, protein is the second most abundant nutrient of qingke grains, representing up to 17.5% of its total content. Owing to its high content and nutritional value, qingke is a promising protein alternative [15]. The grain protein content (GPC) is a crucial quality trait that significantly impacts the functional value of grains [16].

In recent years, the first high-quality reference genome map of qingke has revealed unprecedented possibilities for investigating barley grain development dynamics at the whole-transcriptome scale [17]. Preliminary transcriptomic studies of qingke seeds have revealed critical metabolic networks and mechanisms regulating grain development, such as anthocyanin accumulation [18]. Transcriptomic analysis of barley grain development revealed a gene expression atlas, offering insights into the regulatory dynamics driving grain development. With the increase in the number of qingke cultivars, reports on their nutritional components have also gradually increased. Quantitative proteomic profiling has been conducted, providing insights into the proteins associated with β-glucan accumulation in hulless barley grains [4]. The cultivar-specific differences in protein expression and metabolite accumulation during grain development in important qingke cultivars have not been systematically analyzed. The mechanisms by which these differences influence the formation of final quality traits remain unclear. In particular, few studies have explored the mechanisms of β-glucan (BG), starch, and anthocyanin synthesis and accumulation in qingke cultivars at the metabolite and protein levels.

The combination of metabolomic profiling with proteomic data has emerged as a powerful approach for elucidating complex metabolic networks in plants. The mechanisms of Tibetan hulless barley grain coloration have been clarified [19,20]. In this study, three main cultivars (Dulihuang, Kunlun 14, and Heilaoya) were selected, and seed samples were collected at four key time points (16, 20, 36, and 42 days after flowering (DAF)) during the seed-filling stage. Dulihuang is a traditional Tibetan cultivar, while Kunlun 14, which is cultivated for high-altitude adaptation, exhibits robust stress resistance and stable yield. Heilaoya accumulates anthocyanins and other flavonoids, offering unique antioxidant properties. These cultivars represent divergent genetic backgrounds and metabolic pathways, enabling a comprehensive investigation into the molecular mechanisms underlying grain development and quality formation in qingke. An integrated metabolomics and proteomics analysis was conducted to visualize dynamic changes in seed composition, identify critical pathways and compounds involved in quality formation, and identify critical differentially expressed proteins. These results not only enhance our comprehension of the molecular mechanisms in seed development but also offer valuable insights for future gene function research and targeted breeding strategies aimed at improving qingke grain quality.

## 2. Results

### 2.1. Metabolic Profiles of Qingke with Different Developmental Stages

#### 2.1.1. Metabolite Identification and Analysis

**Figure 1:**
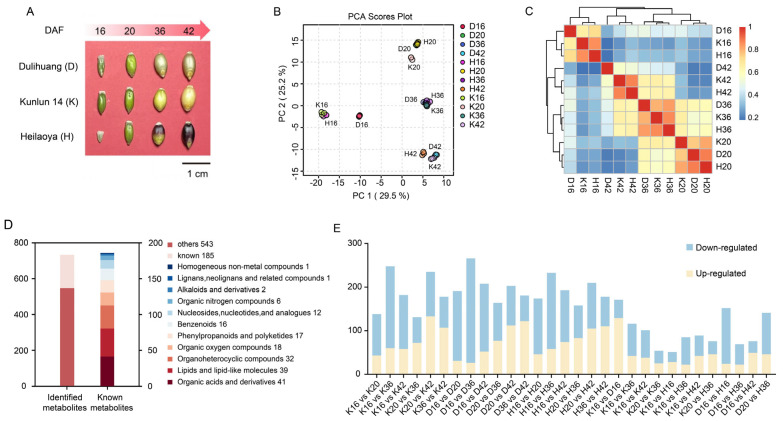
Qingke seed morphology and metabolite analysis at four key developmental stages (I, II, III, and IV): (**A**) Grain phenotype. (**B**) PCA of metabolomic data from four developmental stages and 36 qingke samples. (**C**) Hierarchical clustering analysis of metabolite distribution profiles of Dulihuang, Kunlun 14, and Heilaoya at four developmental stages. The color scale 0−1 represents Spearman’s correlation coefficient. (**D**) Numbers and classification of the identified and annotated metabolites. (**E**) Statistics of differentially accumulated metabolites, including upregulated and downregulated metabolites, in each comparison group.

The phenotypic characteristics of the seeds of three qingke cultivars (Dulihuang, Kunlun 14, and Heilaoya) during four key developmental stages are shown in Figure 1A. In total, 728 metabolites were initially detected via LC-MS (Table S1). The PCA results indicated that PC1 accounted for 29.5%, and PC2 contributed 25.2% of the variance, demonstrating a distinct alteration in metabolite levels across the stages (Figure 1B). Additionally, the close clustering of triplicate samples for each period reflected the high reliability and reproducibility of the experiment. Correlation results revealed that the metabolites were less closely associated with each other across different stages than in the same stage (Figure 1C). The global metabolite accumulation patterns were nonspecific to the three cultivars and related to the developmental phases of the seeds, and the metabolic processes of these three cultivars may be regulated by the same gene expression or metabolic pathways at the same time, resulting in similar metabolic processes. Among the 728 metabolites, 185 were annotated using the HMDB database (Table S2). These metabolites were categorized into 11 distinct biochemical classes (Figure 1D). Among these, organic acids and their derivatives were the most abundant, comprising 41 metabolites, followed by lipids and lipid-like molecules, with 39 metabolites. Other significant categories included 32 organoheterocyclic compounds, 18 organic oxygen compounds, 17 phenylpropanoids and polyketides, and 16 benzenoids. Additionally, 12 nucleotides and derivatives, 6 organic nitrogen compounds, 2 alkaloids and derivatives, 1 lignan and related compounds, and 1 homogeneous nonmetal compound were identified.

#### 2.1.2. Differentially Accumulated Metabolites in Qingke Seed Fractions

In total, 653 differentially accumulated metabolites (DAMs) were identified among the 30 comparison groups (Table S3). The D16 vs. D36, K16 vs. K36, and H16 vs. H36 comparison groups presented the greatest number of DAMs, with 266 (26 upregulated and 240 downregulated), 248 (60 upregulated and 188 downregulated) and 233 (58 upregulated and 175 downregulated) DAMs, respectively. In addition to the developmental stages, the significant differences between cultivars played a major role in the discrimination of metabolic profiles. To better elucidate the varietal differences, we compared the DAMs among three qingke cultivars at the same developmental period. A total of 335, 246, and 331 DAMs were identified across the D vs. K, K vs. H, and D vs. H comparison groups, respectively, suggesting that the cultivar has a weaker influence on metabolism than developmental processes (Figure 1E).

#### 2.1.3. Cluster Analysis of the Accumulation Patterns of Shared DAMs in Dulihuang, Kunlun 14, and Heilaoya

**Figure 2:**
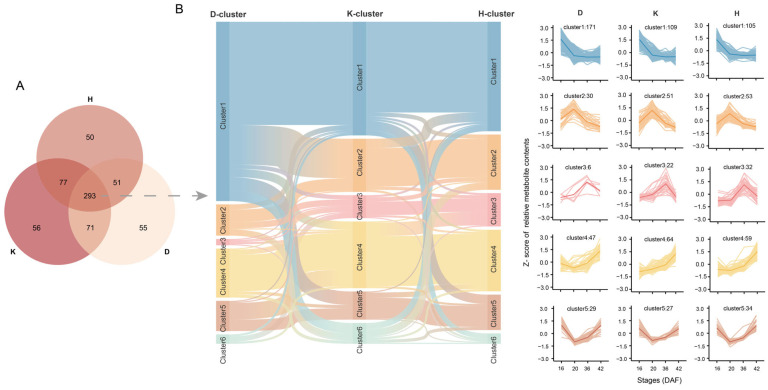
Cluster analysis of the accumulation patterns of the shared DAMs in Dulihuang, Kunlun 14, and Heilaoya: (**A**) Venn diagram of the DAMs in each of the three cultivars. (**B**) Distribution patterns of 293 metabolites at four seed developmental stages.

We identified a total of 470, 497, and 471 DAMs in Dulihuang, Kunlun 14, and Heilaoya, respectively. Through pairwise comparisons across the four developmental stages, 293 DAMs were shared among them (Figure 2A). The main pathways associated with these metabolites included unsaturated fatty acid biosynthesis, linoleic acid metabolism, purine metabolism, and pyrimidine metabolism. To explore the metabolic changes during the development of qingke grains, all 293 proteins were categorized into several clusters on the basis of their accumulation profiles (Figure 2B): highest metabolite content at 16 DAF (the content decreased with development), at 20 DAF (the content first increased but then decreased), and at 36 DAF and at 42 DAF (the content increased with development), and one cluster presented the highest metabolite content at 16 and 42 DAF. In addition, some metabolites did not belong to any of these five categories (Table S4). Most of the 293 metabolites were classified into Cluster 1 (171 for Dulihuang, 109 for Kunlun 14, and 106 for Heilaoya), followed by Cluster 4. Relatively few metabolites were classified in the remaining models (10 for Dulihuang, 20 for Kunlun 14, and 10 for Heilaoya). More than 50% (158 of 293) of the metabolites were classified into the same cluster in Dulihuang and Kunlun 14, and more than two-thirds (206 of 293) of the metabolites shared the same cluster in Kunlun 14 and Heilaoya. We found that the patterns of DEPs between the developmental periods of Kunlun 14 and Heilaoya were similar. Consistent with previous results, the metabolite contents varied more significantly among the stages than among the qingke accessions. There were 83 metabolites that exhibited the same accumulation pattern (belonging to Cluster 1), such as fatty acids and their derivatives (16-hydroxyhexadecanoic acid and alpha-linolenic acid), phenolic compounds (2,4,6-tri-tert-butylphenol, 2-hydroxycinnamic acid, p-coumaraldehyde, fraxin and 4-hydroxybenzaldehyde) and secondary metabolites (quinoline, methyl dihydrojasmonate and avocadyne-1-acetate). Moreover, only 26 metabolites had the same accumulation pattern (belonging to Cluster 4), including procyanidin B1, catechin, and dihydrosphingosine.

### 2.2. Proteomic Profiles of Qingke with Different Developmental Stages

#### 2.2.1. Protein Identification and Analysis

**Figure 3:**
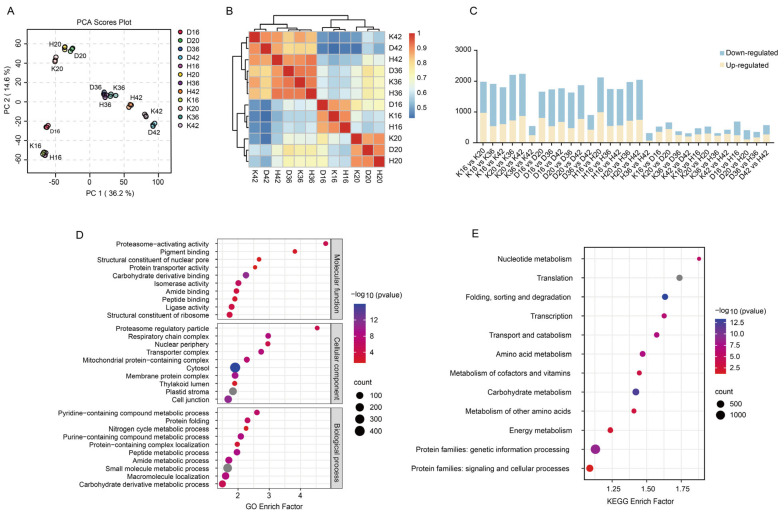
Protein identification and analysis: (**A**) PCA of proteomic data from four developmental stages and 36 qingke samples. (**B**) hierarchical clustering analysis of the protein expression profiles of Dulihuang, Kunlun 14, and Heilaoya at four developmental stages. The color scale 0−1 represents Spearman’s correlation coefficient. (**C**) statistics of the differentially expressed proteins, including upregulated and downregulated proteins, in each comparison group. (**D**) Top 10 GO terms of each category for the recognized proteins in the proteome. (**E**) KEGG pathway enrichment of the identified proteins. The enrichment factor is the percentage of members out of the total number detected. The bubble size represents the number of members detected in the KEGG pathway, and the color of the bubble represents the −log 10 (*p* value).

In the 30 samples, 4864 proteins were identified and quantified by our proteomic data (Table S5). These proteins were distributed mainly across the cell membrane, cytoskeleton, Golgi apparatus, endoplasmic reticulum, mitochondria, chloroplasts, nucleus, and extracellular space. With respect to length, the proteins displayed a wide spectrum of variability (from 20 to 5072). The molecular weights of the proteins had a wide distribution, spanning from 2 to 563 kDa, indicating comprehensive coverage in terms of protein mass. The PCA results revealed that PC1 and PC2 accounted for 36.2% and 14.6% of the variance, respectively, indicating that Kunlun 14 and Heilaoya exhibited a close relationship in the early stages of qingke growth, whereas the three cultivars were relatively consistent at 36 days after flowering, with small differences between the cultivars (Figure 3A). The correlation analysis findings aligned closely with those from the PCA (Figure 3B). Metabolic activities are time-dependent, and during these two periods, the metabolic patterns of qingke may have undergone significant changes, resulting in similar protein expression patterns at the early and late stages of seed development. KEGG pathway analysis revealed significant enrichment in pathways associated with nucleotide metabolism, translation, protein folding, sorting, degradation, transcription, transport, catabolism, amino acid metabolism, cofactor and vitamin metabolism, and carbohydrate metabolism (Figure 3D). The main GO annotations of the molecular functions were proteasome-activating activity (GO:0036402), pigment binding (GO:0031409), structural constituent of the nuclear pore (GO:0017056), structural constituent of nuclear pore protein transporter activity (GO:0140318), and carbohydrate derivative binding (GO:0097367). The main biological processes included the pyridine-containing compound’s metabolic process (GO:0072524), protein folding (GO:0006457), the nitrogen cycle’s metabolic process (GO:0071941), the purine-containing compound’s metabolic process (GO:0072521), protein-containing complex localization (GO:0031503), and the peptide metabolic process (GO:0006518) (Figure 3E).

#### 2.2.2. Differentially Expressed Proteins in Qingke Seed Fractions

A total of 4420 DEPs were identified. At any two time points, 3583, 3795, and 3593 DEPs were identified in Dulihuang, Kunlun 14, and Heilaoya, with 2742 DEPs shared among them (Table S6, Figure 3C). When comparing adjacent time points (for example, stage II vs. stage I), we observed that the smallest variation in differentially expressed proteins occurred between stage IV and stage III, which aligns with the initial correlation analyses. A total of 1476, 1370, and 1566 DEPs were identified across the D vs. K, K vs. H, and D vs. H comparison groups, respectively, which is consistent with the metabolomics results.

#### 2.2.3. PPI Network Construction of the DEPs

**Figure 4:**
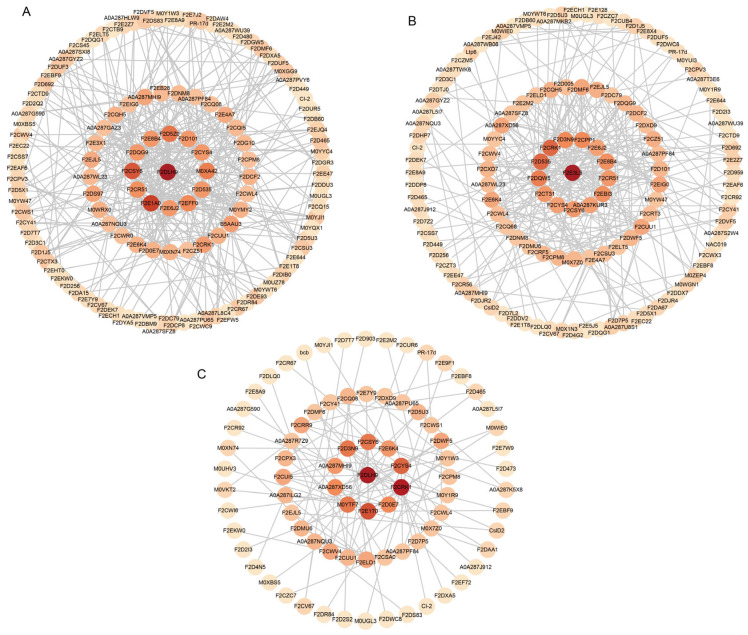
PPI networks of the DEPs: (**A**) Dulihuang vs. Kunlun. (**B**) Dulihuang vs. Heilaoya. (**C**) Kunlun 14 vs. Heilaoya. The circles represent the DEPs in each comparison, and darker colors indicate greater connectivity.

To explore the functions of the DEPs, we constructed PPI networks on proteins with differential expression in the Dulihuang vs. Kunlun 14, Dulihuang vs. Heilaoya, and Kunlun 14 vs. Heilaoya comparison groups (Table S7). As shown in Figure 4A, in the PPI network of the DEPs in the Dulihuang vs. Kunlun 14 comparison, we observed strong connectivity among F2E6J2 (sucrose synthase); A0A287GAZ3 (alpha, alpha-trehalose-phosphate synthase); M0YMY2, F2D0E7, and F2CQH5 (UDP-glucose 6-dehydrogenase); A0A287PF84 and F2CWL4 (trehalose 6-phosphate phosphatase); F2CYS4 (sucrose-phosphate synthase); F2D5Z0 (UDP-glucuronate decarboxylase); and F2E3X1 (predicted protein). These interactions highlight the central role of sucrose synthase in coordinating carbohydrate metabolism, including sucrose and trehalose biosynthesis, nucleotide sugar production, and cell wall polysaccharide synthesis. We also observed strong connectivity among F2DLH9 (predicted protein) and F2D101 (ribosomal protein L19), F2E7Y9 (large ribosomal subunit protein UL18c), F2DUF5 (predicted protein, ribosome biogenesis), F2EB28 (protein transport protein Sec61 subunit beta), and F2ECH1 (predicted protein and proteolysis), which play a role in protein synthesis and modification. In the Dulihuang vs. Heilaoya PPI network (Figure 4B), several hub proteins with functions related to photosynthesis and electron transport, such as F2CRK1 and F2CR92 (predicted protein, photosystem II assembly), F2D535 and F2EBI3 (predicted protein, chlorophyll biosynthetic process), A0A287SFZ8 (thioredoxin domain-containing protein, cytochrome b6f complex assembly), F2D7P5 (plastocyanin), and F2DQW5 (cytochrome b-c1 complex subunit Rieske, mitochondrial), formed strong connections, and their interactions appeared crucial for network stability. In the PPI network of the DEPs in the Kunlun 14 vs. Heilaoya comparison, F2CRK1, A0A287SFZ8, F2CWI6 (predicted protein, photosynthesis), F2DMF6 (predicted protein, carotenoid biosynthetic process), and F2D7P5 and F2E9F1 (chlorophyll a-b binding protein, chloroplastic) had a high degree of connectivity (Figure 4C). These proteins were also mainly associated with photosynthesis and electron transport.

#### 2.2.4. Cluster Analysis of Proteins with Shared Expression Patterns in Dulihuang, Kunlun 14, and Heilaoya

**Figure 5:**
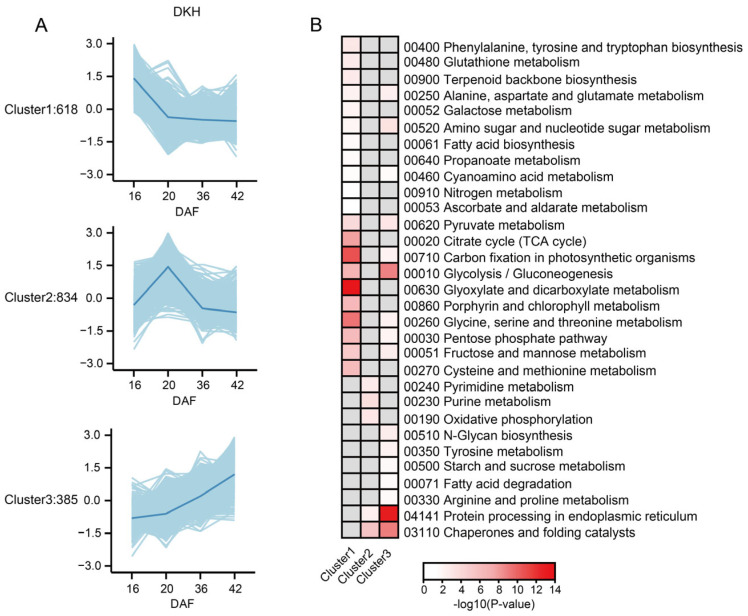
Cluster analysis and functional enrichment of proteins with shared expression patterns in Dulihuang, Kunlun 14, and Heilaoya: (**A**) Major clusters (Clusters 1~3) are shown with light blue trends for proteins with the same trend in Dulihuang, Kunlun 14, and Heilaoya. The average expression levels of genes in each cluster are shown by blue lines. (**B**) Functional category enrichments among the three major clusters are shown in a heatmap. Red, significant enrichment; white, nonsignificant; gray, not detected.

To further elucidate the regulatory networks or signaling pathways critical for the growth and development of qingke, K-means clustering analysis was performed to generate classifiers for 4864 identified proteins (Table S8). As shown in Figure 5A, we identified proteins with consistent expression patterns among the three types of qingke. Cluster 1 included the 618 proteins expressed at the highest level at 16 DAF, which were functionally enriched in carbon fixation in photosynthetic organisms; glycine, serine, and threonine metabolism; glyoxylate and dicarboxylate metabolism; the TCA cycle; glycolysis/gluconeogenesis; and porphyrin and chlorophyll metabolism. In this study, a marked decrease in the abundance of multiple proteins linked to photosynthesis and electron transport was observed. The reduction in the abundance of photosynthetic proteins aligns with the gradual de-greening of seeds following time point II (Figure 5B). In particular, the abundance of several photosynthetic proteins in Cluster 1, including photosystem I reaction center subunit III (F2DUW3), photosystem I P700 chlorophyll a apoprotein A1 (A0A218LNC7), and predicted proteins (F2CR92, F2CWI6) involved in photosystem I function, progressively decreased throughout development. Additionally, a predicted protein (F2D6M2), which is implicated in photosynthetic electron transport within photosystem I, also exhibited significantly decreased abundance. Similarly, proteins related to cellular energy metabolism, including ATP synthase subunit beta (A0A218LNG7) and chloroplastic ATP synthase epsilon chain (ATP synthase F1 sector epsilon subunit, A0A218LNA7), decreased significantly in abundance throughout seed maturation. A reduction in the abundance of specific electron transport proteins, including ferredoxin--NADP reductases (A0A8I7BCY1); ferredoxin (F2D976); predicted protein (F2EF72, with plastocyanin-like domain); cytochrome b6 (A0A218LNJ7); cytochrome f (A0A218LND2); and (S)-2-hydroxy-acid oxidase, FMN-dependent alpha-hydroxy acid dehydrogenase family (A0A8I6XTA8), was observed. Overall, the coordinated downregulation of photosynthetic, electron transport, and energy metabolism proteins reflected a metabolic shift from active photosynthesis to storage compound accumulation and the continuous maturation of seeds. This observation is consistent with the phase marking the initiation of cell expansion and dry matter accumulation, during which large amounts of energy are required [21].

A total of 834 proteins with the highest expression at 20 DAF in Cluster 2 were functionally enriched in chaperones and folding catalysts, purine metabolism, oxidative phosphorylation, pyrimidine metabolism, and protein processing in the endoplasmic reticulum, which is consistent with the physiological changes observed at 20 DAF. Qingke enters the early stage of storage material accumulation, during which the cells actively proliferate and synthesize a large number of proteins and nucleotides, providing the material foundation for subsequent seed maturation. Finally, Cluster 3 included 385 proteins that were specifically expressed at late stages and enriched in protein processing in the endoplasmic reticulum, chaperone and folding catalysts, glycolysis/gluconeogenesis, amino sugar and nucleotide sugar metabolism, pyruvate metabolism, and fructose and mannose metabolism. Notably, three thioredoxin domain-containing proteins (A0A8I7BH42, A0A8I6WND3, and F2E1T7) from the protein disulfide isomerase family, which may play critical roles in protein folding and redox regulation during seed maturation, were identified.

#### 2.2.5. Cluster Analysis of Proteins with Pairwise-Shared Expression Profiles in Dulihuang, Kunlun 14, and Heilaoya

To identify the proteins associated with specific physiological traits of particular varieties, we performed comparative proteomic analysis to reveal key proteins that exhibited conserved expression patterns in the two varieties but differed significantly in the third. The proteins that exhibited similar expression patterns in Dulihuang and Kunlun 14 presented distinct expression profiles in Heilaoya and were functionally enriched in protein processing in the endoplasmic reticulum, exosomes, and membrane trafficking. The classification is shown in Table S8. Interestingly, the proteins expressed at the highest level at 16 DAF in Dulihuang and Kunlun 14 were found to be delayed in expression in Heilaoya and were functionally enriched in amino sugar and nucleotide sugar metabolism through the MAPK signaling pathway, which is involved in plant and protein processing in the endoplasmic reticulum. This delayed expression in Heilaoya coincides with the significantly smaller grain size observed at 16 DAF compared with Dulihuang and Kunlun 14 (Figure S1). Among these proteins, UDP-arabinopyranose mutase (A0A0U2GJ84) is essential for the interconversion of UDP-arabinofuranose and UDP-arabinopyranose, which are key substrates for cell wall biosynthesis. The delayed activation of this enzyme in Heilaoya may impact early grain development by affecting cell wall integrity and expansion. Additionally, the involvement of these proteins in amino sugar and nucleotide sugar metabolism suggests potential effects on polysaccharide synthesis and cellular structure formation. The MAPK signaling pathway is essential for cell proliferation and stress responses, while disruptions in protein processing in the endoplasmic reticulum may affect proper protein folding and transport, further influencing grain filling and growth.

Proteins that exhibited similar expression patterns in Heilaoya and Dulihuang presented distinct expression profiles in Kunlun 14 and were functionally enriched in carbon fixation in photosynthetic organisms, cysteine and methionine metabolism, and the pentose phosphate pathway. Proteins that exhibited similar expression patterns in Heilaoya and Dulihuang presented distinct expression profiles in Kunlun 14 (some proteins were highly expressed in Kunlun 14) and were functionally enriched in carbon fixation in photosynthetic organisms, the pentose phosphate pathway, and cysteine and methionine metabolism. These metabolic adjustments enhance photosynthetic efficiency under low CO_2_ conditions, bolster antioxidant capacity against intense UV radiation, and optimize carbon allocation for stress resilience, which may match its high-altitude adaptability. Proteins with similar expression patterns in Kunlun 14 and Heilaoya but distinct in Dulihuang were functionally enriched in the TCA cycle and spliceosome. In conclusion, Heilaoya exhibited delayed activation of grain development proteins (e.g., cell wall biosynthesis), correlated with its smaller grain size at the early stage, while Kunlun 14 showed unique metabolic emphasis (carbon fixation, pentose phosphate pathway), potentially enhancing stress resilience. Dulihuang diverged in energy metabolism (TCA cycle) and RNA processing, suggesting alternative regulatory strategies.

## 3. Discussion

### 3.1. Metabolites and Proteins Involved in Energy Metabolism and Carbohydrate Metabolism

**Figure 6:**
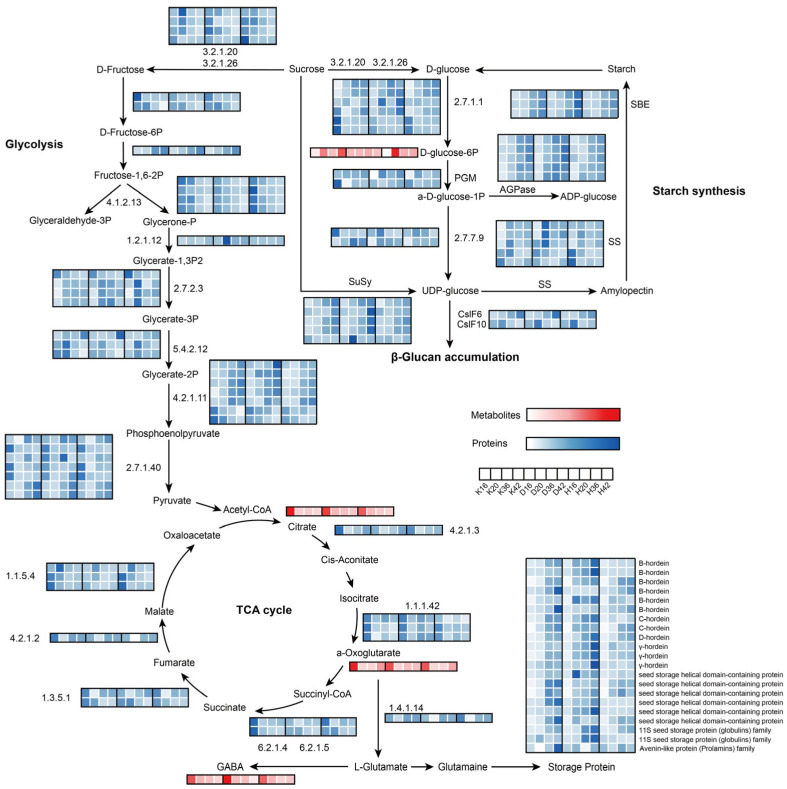
Visualization of the metabolites and proteins in biochemical pathway maps related to carbohydrate metabolism and energy metabolism in developing qingke seeds. Z score fold change values are shown on a color scale that is proportional to the abundance of each metabolite and protein. 3.2.1.20, maltase-glucoamylase; 3.2.1.26, beta-fructofuranosidase; 4.1.2.13, fructose-bisphosphate aldolase; 1.2.1.12, glyceraldehyde 3-phosphate dehydrogenase; 2.7.2.3, phosphoglycerate kinase; 5.4.2.12, 2,3-bisphosphoglycerate-independent phosphoglycerate mutase; 4.2.1.11, phosphopyruvate hydratase; 2.7.1.40, pyruvate kinase; 4.2.1.3, aconitate hydratase; 1.1.1.42, isocitrate dehydrogenase; 6.2.1.4, 6.2.1.5, succinyl-CoA synthetase alpha subunit; 1.3.5.1, succinate dehydrogenase; 4.2.1.2, fumarate hydratase; 1.1.5.4, malate dehydrogenase; 2.7.1.1, hexokinase; PGM, phosphoglucomutase; AGPase, ADP-glucose pyrophosphorylase; 2.7.7.9, UDP-glucose pyrophosphorylase; CslF6, cellulose synthase-like F6; CslF10, cellulose synthase-like F10; SS, starch synthase; SBE, starch branching enzyme.

The selected phase (16–42 days after flowering) corresponds to the grain-filling stage, a critical developmental stage characterized by the active synthesis and substantial accumulation of starches and storage proteins until filling is essentially complete, which is followed by dehydration and drying. To explore metabolic pathway dynamics during grain development, we initially examined the differential regulation of metabolites and proteins related to carbon and energy metabolism pathways among the three colored qingke cultivars. Carbon and energy metabolism play a central role in seed development [22]. During grain development, the coordinated activities of photosynthesis, glycolysis, the tricarboxylic acid (TCA) cycle, and the oxidative pentose phosphate (OPPP) pathway ensure the supply of carbon skeletons and reducing equivalents [23]. These metabolic processes not only support the synthesis and regeneration of essential cofactors but also maintain the metabolic flux necessary for the production of sucrose, starch, and storage proteins [24]. The expression levels of the enzymes involved and the accumulation levels of the metabolites are shown in Figure 6. We found that the predicted proteins (F2CW75 and F2DNJ1), mitochondrial electron transport proteins such as the cytochrome b-c1 complex subunit Rieske (F2CYU4 and F2DQW5), malic enzymes (F2DIS4 and F2ELT5), isopropyl malate dehydrogenase-like domain-containing protein (A0A287U3J5), acetyltransferase component of the pyruvate dehydrogenase complex (A0A8I7BCX4) and pyruvate dehydrogenase E1 component subunit alpha (F2DKD7) were significantly upregulated from time points III to IV, suggesting their involvement in aerobic respiration or the regulation of metabolic intermediates to supply reducing equivalents necessary for BG synthesis following seed de-greening.

Starch consists of two glucose polymers, amylose straight-chain starch and amylopectin branched chain starch [25]. Amylose in cereal endosperm is synthesized by GBSSI, whereas several types of enzymes are involved in the amylopectin biosynthetic pathway: AGPase, SS, SBE, and DBE [26,27]. These enzymes play distinct roles but presumably function as part of an interconnected network. In this synthesis network, proteins regulating amylose and amylopectin synthesis may interact. The abundance of starch synthesis-related proteins progressively increased from 16 to 42 DAF, with sucrose synthesis-associated enzymes exhibiting a comparable accumulation pattern during this period. In particular, ADP-glucose pyrophosphorylase (AGPase) was upregulated specifically from time points II to III, which aligns with previous results showing that starch synthesis peaked during the late storage phase of seed development. Interestingly, the abundances of AGPase, sucrose synthase (SuSy), and starch-branching enzyme (SBE) in Dulihuang were significantly greater than those in Kunlun 14 and Heilaoya at 42 DAF, indicating variations in starch synthesis among the three cultivars. The abundance of starch synthase was significantly greater during the early storage phase of seed development. Overall, the observed high enzymatic activity of starch synthesis-related proteins in Dulihuang during the late storage phase suggests a robust starch biosynthesis pathway. This may result in greater starch accumulation and a unique starch composition, such as an increased proportion of amylopectin. A relatively high amylopectin content may improve starch functional properties, including gelatinization and digestibility [28], making Dulihuang a promising candidate for high-starch yield applications in food. Specifically, in Dulihuang, starch synthase isoforms (A0A8I6WE99, A0A0U2S9G2, and Q8H1Y7) were more abundant at 20 DAF than in Kunlun 14 and Heilaoya, whereas in Heilaoya, isoforms (C3W8L3 and F2DWF5) peaked at 16 DAF, surpassing both Kunlun 14 and Dulihuang. The high abundance of starch synthase during 16–20 DAF is closely associated with key developmental processes, including cell wall thickening, the deposition of large starch granules, and a clear differentiation of aleurone cells. This phase marks a critical period in seed development, where increased starch synthase activity drives rapid starch accumulation, providing the energy reserves necessary for seed maturation.

Members of two cellulose synthase-like subfamilies (CslF and CslH), known for their role in β-glucan synthesis, have been well documented [29]. CslF6 levels at various stages of grain development may strongly affect β-glucan accumulation in barley grains. CslF6 has been reported to be dose-dependent in β-glucan biosynthesis, with endosperm-specific overexpression resulting in substantial increases in β-glucan content in barley, wheat, and rice grains [30,31]. In this study, the abundance of CslF10 slightly increased from 16 to 20 DAF but then decreased moderately after 20 DAF, whereas the abundance of CslF6 increased. The abundance of CslF10 was not different, whereas Heilaoya presented relatively lower amounts of CslF6 at 36 and 42 DAF than Kunlun 14 and Dulihuang did, which may suggest a possible link to β-glucan biosynthesis, though this causal relationship requires direct experimental validation through targeted approaches such as gene knockouts or enzyme assays. Qingke storage protein is a complex mixture of different components. The major endosperm storage proteins of barley are alcohol/water-soluble prolamins, called hordeins. On the basis of their sequence relationships, cereal prolamins are further classified into high-molecular-weight (HMW) prolamins (D hordein), sulfur-poor prolamins (C-hordein), and sulfur-rich prolamins (γ- and B-hordein). These storage proteins presented roughly the same accumulation trends during grain maturity in the three cultivars, with gradually increasing content, resembling the changes observed in wheat storage proteins during the grain-filling stage [32]. In addition, we found that the final accumulation of the vast majority of storage proteins in Dulihuang and Kunlun 14 was much greater than that found in Heilaoya. For γ-hordein, there was an approximately 2-fold difference in content between Dulihuang and Kunlun 14 and more than a 2.5-fold difference in content between Dulihuang and Heilaoya at 42 DAF.

### 3.2. Metabolites and Proteins Involved in Flavonoid Biosynthesis

**Figure 7:**
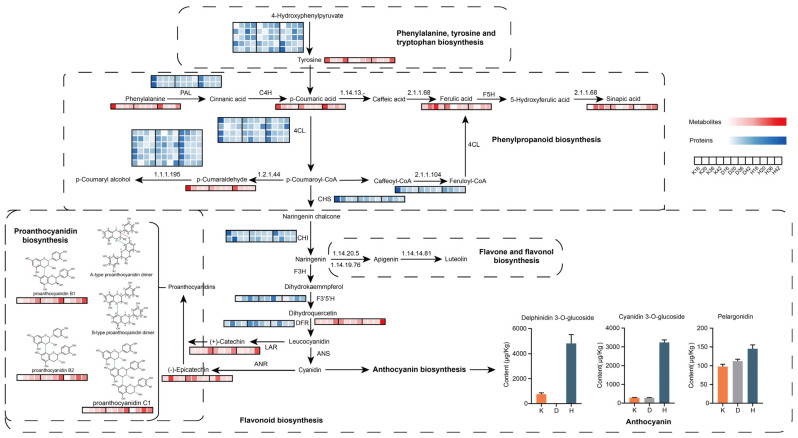
Visualization of the metabolites and proteins in biochemical pathway maps related to flavonoid biosynthesis in developing qingke seeds. The heatmap was plotted using normalized expression values of metabolites and proteins with Z scores. Total anthocyanin content in the three cultivars at 42 DAF. PAL, phenylalanine ammonia-lyase; C4H, 4-hydroxylase; 4CL, CoA ligase; 1.2.1.44, cinnamoyl-CoA reductase; 1.1.1.195, cinnamyl-alcohol dehydrogenase; CHS, chalcone synthase; CHI, chalcone isomerase; F3H, flavanone 3-hydroxylase; F3′5′H, flavonoid 3′,5′-hydroxylase; DFR, dihydroflavonol-4-reductase; LAR, leucoanthocyanidin reductase; ANS, anthocyanidin synthase; ANR, anthocyanidin reductase; 1.14.13.-, p-coumaroyl-CoA: caffeoyl-CoA 3-hydroxylase; 2.1.1.68, caffeic acid 3-O-methyltransferase; F5H, flavanone 5- hydroxylase; 2.1.1.104, caffeoyl-CoA O-methyltransferase; 1.14.20.5, flavone synthase I; 1.14.19.76, flavone synthase II; 1.14.14.81, flavonoid 3′,5′-hydroxylase.

As a crop predominantly cultivated in the extreme conditions of the Tibetan Plateau (altitudes > 4000 m), qingke has evolved under distinctive selective forces, including strong UV-B radiation, low temperatures, and low barometric pressure [33]. Hulless barley has developed multiple adaptive mechanisms to mitigate the detrimental effects resulting from these pressures. One of the central components of the hierarchical metabolic response involves the upregulation of flavonoid and anthocyanin synthesis as antioxidant defenses [34]. Flavonoids are a large category of polyphenolic secondary metabolites prevalent in plants, contributing to plant growth and development and having prominent food and medical applications. This group includes flavones, flavonols, flavanols/proanthocyanidins, isoflavones and a multitude of minor subclasses, and the most familiar flavonoids are undeniably anthocyanins [35,36]. Anthocyanins are synthesized through the phenylpropanoid and flavonoid pathways in the cytoplasm, and the final products are transported into the plant vacuole. The biosynthetic pathway originates from phenylalanine, where p-coumaroyl-CoA serves as a precursor. This process is mediated by the enzymatic actions of phenylalanine ammonia-lyase (PAL) and 4-coumarate-CoA ligase (4CL), directing metabolic flow into the flavonoid biosynthesis pathway (4CL). We found that the abundances of these two gateway enzymes decreased during seed ripening, which was consistent with the changes in the contents of phenylpropanoids, such as p-coumaric acid and p-coumaraldehyde (Figure 7). A single p-coumaroyl-CoA and three molecules of malonyl-CoA are converted to produce a yellow chalcone through the enzyme chalcone synthase (CHS) (A0A8I6W949) [37]. Only one corresponding protein was found in our dataset, which exhibited a similar regulatory pattern, i.e., decreasing abundance with increasing maturity. Chalcone is converted to naringenin by chalcone isomerase (CHI) and subsequently hydroxylated at the C3 position with the participation of flavanone-3-hydroxylase (F3H). The conversion of dihydrokaempferol into dihydroquercetin is catalyzed by flavonoid 3′,5′-hydroxylase (F3′5′H). The peak abundances of CHI proteins were primarily observed at 16 or 20 days after flowering, while the content in Dulihuang was significantly lower than that in the other two varieties. F3′5′H showed a significant increase from 20 DAF onwards and reached a peak of approximately 2-fold. The third stage is the anthocyanin biosynthesis stage, in which dihydroflavonols are catalyzed by dihydroflavonol-4-reductase (DFR) to produce leucocyanidins, which are then further catalyzed by enzymes involved in anthocyanin biosynthesis and modification pathways to form colored anthocyanins. DFR was also significantly downregulated in the three colored cultivars at the grain color-changing and maturation stages. In summary, the general tendency of these positive regulatory enzymes involved in flavonoid-derived compound biosynthesis was attenuated with increasing seed maturity at the proteome level, confirming our metabolic findings.

The biosynthesis of proanthocyanidins (PAs) is a branch of the flavonoid pathway, which extends from the central phenylpropanoid metabolic pathway [38]. The fundamental structural units of PAs are (+)-catechin and (−)-epicatechin, which exhibit 2,3-trans and 2,3-cis stereochemical configurations [39]. Among the three cultivars, dihydroquercetin and (+)-catechin reached their highest levels at 42 DAF, with the most pronounced accumulation observed in Heilaoya. Furthermore, another peak was observed at 16 DAF in Dulihuang. (−)-Epicatechin accumulated until 20 DAF and greatly decreased in abundance at the later stages, whereas two proanthocyanidin dimers (procyanidin B1 and B2) and the proanthocyanidin trimer procyanidin C1 identified in our study accumulated more slowly than (−)-epicatechin did, peaking in the later stages. We found a greater accumulation of procyanidin B1, B2, and procyanidin C1 in Dulihuang and Heilaoya than in Kunlun 14, which may imply that these two cultivars have superior antioxidant properties and stress resistance, potentially increasing their nutritional value. Interestingly, we found that several members could be functionally classified as the same enzyme, possibly because they originate from specific gene families or distinct alternative splicing transcripts [40]. The specificity of an individual enzyme or the divergence of activity among multiple copies of the same gene may influence the composition of flavonoid intermediates available for proanthocyanidin (PA) biosynthesis [41].

Numerous studies have focused on the anthocyanin composition of barley grains [42,43,44]. The biosynthesis of anthocyanins appeared to be unaffected by that of proanthocyanidins. Studies in purple waxy hulless barley have demonstrated that both proanthocyanidins and anthocyanins are synthesized through the leucocyanidin pathway but exhibit distinct temporal and spatial accumulation patterns [45]. The targeted metabolomics studies indicated significant differences in anthocyanin accumulation among the three qingke cultivars at maturation, and substantially greater total anthocyanin content was observed in the Heilaoya cultivar than in the other two cultivars, specifically, the three anthocyanins delphinidin 3-O-glucoside (the main anthocyanin in black barley), cyanidin 3-O-glucoside, and pelargonidin, highlighting the potential of this cultivar as a high-anthocyanin germplasm resource [46]. Furthermore, the metabolomics data revealed a significant accumulation of a flavonoid pathway precursor (dihydroquercetin) in the colored cultivars, specifically, in black accessions, during the grain color transition and maturation stages, implying that the increase in flavonoid metabolic flux may play a pivotal role in grain color formation in qingke, which is consistent with findings in Tibetan hulless barley, maize, and peanut exhibiting remarkable anthocyanin accumulation [47,48].

While this study provides comprehensive insights into the metabolic and proteomic dynamics during Tibetan hulless barley grain development, several limitations should be acknowledged. Although we successfully identified 185 metabolites, the majority (543 metabolites) remain unannotated due to limitations in current metabolomics databases and reference standards. These unidentified features represent potential novel metabolites or modified compounds requiring further investigation through advanced analytical techniques and expanded spectral libraries. Our conclusions are primarily based on correlative omics data, which, despite revealing robust associations between metabolites/proteins and developmental stages, do not establish causal relationships. Functional validation (e.g., gene knockout or overexpression) is needed to confirm the roles of key candidates like CslF6 in β-glucan biosynthesis. Furthermore, potential batch effects or environmental variations during sample collection across different stages, though minimized by standardized protocols, may introduce uncontrolled biases. Future studies incorporating controlled growth conditions and larger sample sizes could mitigate such confounding factors. Lastly, the absence of in vitro or in vivo functional follow-up experiments limits mechanistic interpretations. We plan to address these limitations in future work, which will strengthen the translational relevance of our findings for barley breeding programs.

## 4. Materials and Methods

### 4.1. Plant Materials

Three representative qingke cultivars, Dulihuang (D, a yellow-grained cultivar), Kunlun 14 (K, a yellow-grained cultivar), and Heilaoya (H, a black-grained cultivar), were selected for this study based on their distinct phenotypic traits and nutritional profiles. All cultivars of qingke grains were field-grown in Xining, China, from May to August. The plants were tagged when flowering, and the tagged spikes were collected at 16 (I), 20 (II), 36 (III), and 42 (IV) days after flowering (DAF). These stages represent the gradual maturation of qingke, spanning early grain filling to late maturation. Caryopses used for metabolomic and proteomic analyses were rapidly frozen using liquid nitrogen to halt all enzymatic processes and then stored at −80 °C until subsequent metabolite and protein extraction. We used three biological replicates for each developmental stage.

### 4.2. Metabolomic Analysis

Metabolomic analysis was conducted by Qingdao Standard Testing Co., Ltd (Qingdao, China). For the preparation of qingke samples, 100 mg of evenly mixed sample was weighed and dissolved in a 2 mL centrifuge tube. Then, 1 mL of 70% methanol and 3 mm steel balls were added, and the samples were shaken and crushed using an automatic sample rapid grinder (jxfstprp-48, 70 Hz) (Shanghai Jingxin Industrial Development Co., Ltd., Shanghai, China) for 3 min; after cooling, cryogenic sonication (40 kHz) was performed for 10 min. The mixture was centrifuged at 12,000 rpm for 10 min at 4 °C, and the supernatant was diluted 2- to 100-fold. Finally, on-machine inspection was performed with a 0.22 μm PTFE filter head. LC-MS data acquisition was carried out on a Thermo Vanquish Q Exactive HF instrument (Thermo Scientific, Waltham, MA, USA). The MS settings for both positive and negative modes were as follows: heater temperature, 325 °C; sheath gas flow, 45 arbs; aux gas flow, 15 arbs; sweep gas flow, 1 arb; electrospray voltage, 3.5 kV; capillary temperature, 330 °C; and S-Lens RF level, 55%. Compound Discoverer 3.3 (Thermo Scientific, Waltham, MA, USA) was used for processing the MS data, including retention time correction, peak recognition, extraction, alignment, and integration. The resulting data matrix, containing the retention time, molecular weight, and peak intensity, was then analyzed using online and local databases such as Thermo mzCloud, Thermo mzValut (Thermo Scientific, Waltham, MA, USA), and ChemSpider (Royal Society of Chemistry, RSC) for metabolite identification.

### 4.3. Proteomic Analysis

We selected an appropriate method for protein extraction based on the samples, followed by precipitation and purification. We mixed the protein with reducing agent buffer, followed by a reaction at 37 °C for 1 h. Subsequently, iodoacetamide (IAA) was introduced to reach 50 mM final concentration, and the mixture was incubated in the dark for 45 min at room temperature. To quench the IAA reaction, a 1 M dithiothreitol (DTT) solution was added. The solution was adjusted to a final volume of 50 mM NH_4_HCO_3_ to ensure that the urea concentration was less than 1 M. Next, sequencing-grade trypsin solution was added, and digestion was carried out overnight at 37 °C. After digestion, the peptide fragments were desalted using a C18 solid-phase extraction column. The peptides were dissolved in mobile phase A for liquid chromatography and then separated using a high-performance liquid chromatographer system (EASY-nLC 1200, Thermo Fisher Scientific, Waltham, MA, USA) operating at 300 nL/min. Mobile phase A was an aqueous solution containing 0.1% (*v*/*v*) formic acid, while mobile phase B was acetonitrile containing 0.1% (*v*/*v*) formic acid.

Following separation via ultrahigh-performance liquid chromatography, the peptides were ionized using the NSI ion source and analyzed with a Q Exactive HF mass spectrometer (Thermo Scientific, Waltham, MA, USA). The ion source voltage was maintained at 2.3 kV, and both precursor ions and their fragments were analyzed using a high-resolution Orbitrap. The full scan range covered 400 to 1800 *m*/*z* at a resolution of 60,000, while the secondary scan resolution was set to 15,000. Data were collected in data-dependent acquisition (DDA) mode, selecting the top 20 most intense precursor ions for fragmentation in the HCD collision cell at 28 eV. The secondary mass spectrometry analysis was performed in the same manner. To increase efficiency, the parameters used were as follows: automatic gain control (AGC) at 3 × 10^6^, a signal threshold of 10,000 ions, a maximum ion injection time of 50 ms, and a dynamic exclusion time of 45 s to prevent repeated precursor ion scanning. Protein fragmentation simulation and theoretical peptide matching were performed using Proteome Discoverer 2.5. On the basis of the species-specific protein database, protein identification was carried out.

### 4.4. Bioinformatic Analysis

We replaced the missing values in the original abundance data for metabolites and proteins with half the minimum of the non-missing values. Unsupervised principal component analysis (PCA) was conducted using MetaboAnalyst 6.0. Log transformation (log2) and mean centering were applied before PCA. Spearman’s correlation algorithm was used to construct hierarchical clustering (HCL) using R software (version 4.3.2) with the default parameters [49]. HLC was performed via normalization of metabolite and protein expression values with Z scores. Differentially accumulated metabolites (DAMs) were screened for a *p* value < 0.05, FC < 2 or <0.5, and variable importance (VIP) > 1 [50]. The VIP values extracted from the OPLS-DA results were generated using SIMCA software (version 14.1), with default settings [51]. The identification standard for differentially expressed proteins (DEPs) was a protein with a *p* value < 0.05 and a more than 2-fold change (≥2.0 or ≤0.5) [52]. Venn diagrams were used to show the number of DAMs and DEPs. We used Flourish, an online tool (https://flourish.studio/, accessed on 3 January 2025), to create a Sankey diagram. The Gene Ontology (GO) database and the Kyoto Encyclopedia of Genes and Genomes (KEGG) database were used to annotate and classify functions. K-means clustering was applied to visualize metabolites and proteins with similar accumulation patterns using MEV software (version 4.9.0). To explore the functional interactions among the identified proteins, protein-protein interaction (PPI) analysis was conducted using STRING (https://cn.string-db.org/) and Cytoscape software (version 3.10.1) [53].

## 5. Conclusions

In this study, a total of 728 metabolites and 4864 proteins were identified in three qingke varieties across four developmental stages. Comparative analysis of DAMs and DEPs, as well as correlation analyses, revealed that metabolites and proteins exhibited greater stage-specific variation (i.e., among 16, 20, 36, and 42 DAF) than among the qingke accessions. Despite the high similarity observed in the filling process, some quality-related traits exhibited differences. Compared with the other two cultivars, Heilaoya had higher anthocyanin contents. The greater abundance of starch synthesis-related proteins may contribute to increased starch accumulation in Dulihuang and the formation of a greater proportion of amylopectin than in Kunlun 14 and Heilaoya. Integrated multiomics analysis enabled us to systematically reveal the dynamic changes in protein expression profiles and metabolite accumulation during grain development across different qingke cultivars, providing crucial insights into the molecular mechanisms underlying qingke grain development.

## Data Availability

The raw metabolomic data have been uploaded to Metabolight (ID: MTBLS12313). The mass spectrometry proteomics data have been deposited to the ProteomeXchange Consortium (http://proteomecentral.proteomexchange.org) via the iProX partner repository with the dataset identifier PXD061906.

## Supplementary Materials

The following supporting information can be downloaded at: https://www.mdpi.com/article/10.3390/plants14131946/s1.
