# Supplementary material for: Integrated Metabolomics and Proteomics Analyses of the Grain-Filling Process and Differences in the Quality of Tibetan Hulless Barleys
Source: Plants (Basel). 2025 Jun 25;14(13):1946. doi: 10.3390/plants14131946 (PMC12251650; doi:10.3390/plants14131946)
Supplement: Supplementary file 1 [file plants-14-01946-s001.zip › Supporting information/Supplementary Figure/Figure S1.pdf]

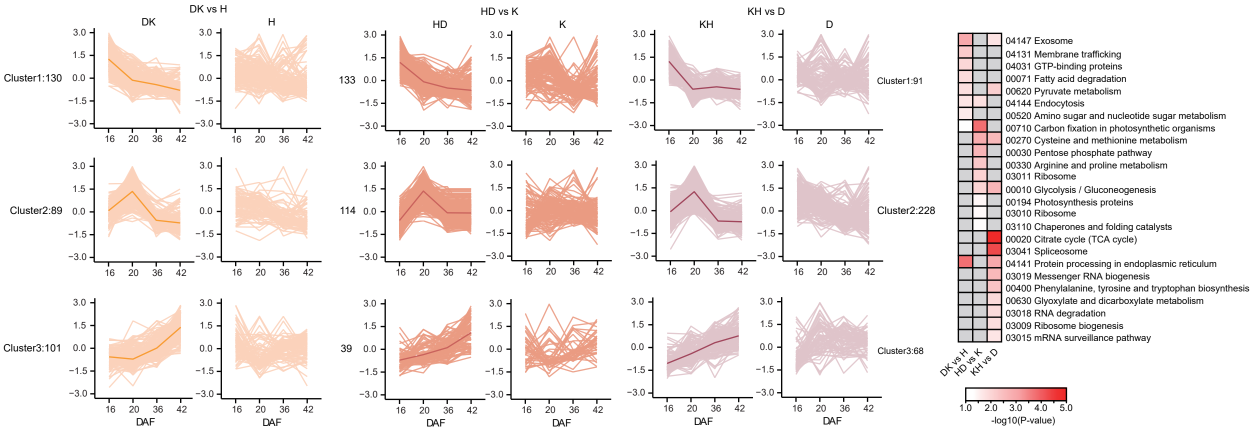


Fig. S1. Cluster analysis of proteins with pairwise-shared expression profiles in Dulihuang, Kunlun 14, and Heilaoya. Three protein categories were identified: Dulihuang & Kunlun 14 vs. Heilaoya, Heilaoya & Dulihuang vs. Kunlun 14, and Kunlun 14 & Heilaoya vs. Dulihuang. Each category (light orange, light brown, and light pink) presented similar trends between the two varieties but differed from the third and was further divided into three clusters.
